# The β-adrenergic receptor blocker and anti-inflammatory drug propranolol mitigates brain cytokine expression in a long-term model of Gulf War Illness

**DOI:** 10.1016/j.lfs.2021.119962

**Published:** 2021-09-24

**Authors:** Lindsay T. Michalovicz, Kimberly A. Kelly, Diane B. Miller, Kimberly Sullivan, James P. O’Callaghan

**Affiliations:** a Health Effects Laboratory Division, Centers for Disease Control and Prevention-National Institute for Occupational Safety and Health, Morgantown, WV, USA; b School of Public Health, Boston University, Boston, MA, USA

**Keywords:** Gulf War Illness, Neuroinflammation, Propranolol, Treatment, Corticosterone, Diisopropyl fluorophosphate

## Abstract

**Aims:**

Growing evidence suggests that Gulf War Illness (GWI) is the result of underlying neuroimmune dysfunction. For example, previously we found that several GWI-relevant organophosphate acetylcholinesterase inhibitors produce heightened neuroinflammatory responses following subchronic exposure to stress hormone as a mimic of high physiological stress. The goal of the current study was to evaluate the potential for the β-adrenergic receptor inhibitor and anti-inflammatory drug, propranolol, to treat neuroinflammation in a novel long-term mouse model of GWI.

**Main methods:**

Adult male C57BL/6J mice received a subchronic exposure to corticosterone (CORT) at levels mimicking high physiological stress followed by exposure to the sarin surrogate, diisopropyl fluorophosphate (DFP). These mice were then re-exposed to CORT every other week for a total of five weeks, followed by a systemic immune challenge with lipopolysaccharide (LPS). Animals receiving the propranolol treatment were given a single dose (20 mg/kg, i.p.) either four or 11 days prior to the LPS challenge. The potential anti-neuroinflammatory effects of propranolol were interrogated by analysis of cytokine mRNA expression.

**Key findings:**

We found that our long-term GWI model produces a primed neuroinflammatory response to subsequent immune challenge that is dependent upon GWI-relevant organophosphate exposure. Propranolol treatment abrogated the elaboration of inflammatory cytokine mRNA expression in the brain instigated in our model, having no treatment effects in non-DFP exposed groups.

**Significance:**

Our results indicate that propranolol may be a promising therapy for GWI with the potential to treat the underlying neuroinflammation associated with the illness.

## 1. Introduction

Neuroinflammation is of increasing interest with respect to acute and persistent neurological illness and disease. A growing body of research has supported a causal role for prolonged neuroimmune dysfunction in Gulf War Illness (GWI), a multi-symptom illness experienced by nearly one-third of veterans of the 1990–91 Gulf War [1–30]. There is significant evidence that GWI is the result of toxic chemical exposures and adverse conditions experienced by soldiers during their deployment [31–34]. While Gulf War veterans had the potential for exposure to several toxic agents, pesticides and chemical weapons continue to be the most widely studied and supported initiating exposures associated with the development of GWI [32,33,35]. To understand how these combinations of exposures may have instigated illness among the veterans of the Gulf War, we developed an animal model of GWI that combines exposure to GWI-relevant organophosphate neurotoxicants, such as the nerve agent sarin, its surrogate, diisopropyl fluorophosphate (DFP), or the pesticide chlorpyrifos, in combination with prior exogenous exposure to the stress hormone corticosterone (CORT) at levels mimicking high physiological stress for several days [1,7,10,12,13,36]. We have found that this exposure model instigates robust neuroinflammation in rodents. Moreover, the neuroinflammatory changes that have been identified in animal models and ill veterans can be connected to the multitude of different body system symptoms experienced by veterans with GWI, such as cognitive dysfunction, memory deficit, chronic pain, and gastrointestinal distress, through their similarities with sickness behavior, which is driven by neuroinflammation [37]. Thus, targeting the underlying neuroimmune dysfunction therapeutically carries the potential to not only alleviate multiple symptoms associated with GWI, but also treat the underlying cause of the illness.

As the role of neuroinflammation in disease has been uncovered, there has been significant interest in the potential for anti-inflammatory therapeutics to treat neurological disease. Recently, the β-adrenergic receptor blocker, propranolol, has been demonstrated to have anti-neuroinflammatory effects. Treatment with propranolol has been shown to reduce microglial activity and brain inflammation in several animal models of neurological disorders [38–41]. Several studies have suggested that β-adrenergic signaling is significant to the induction of inflammatory cytokines in the brain both by central and peripheral signaling mechanisms [42–44]. As dysfunction in β-adrenergic signaling has been implicated in the etiology of GWI [45–47], and found alongside aberrant neuroimmune signaling, propranolol provides an attractive candidate for the treatment of GWI.

In this study, we expanded upon our previously published acute, in theatre GWI mouse model to produce a long-term mouse model by introducing intermittent re-exposure to CORT following GWI initiation with combined CORT DFP exposure, capturing a timepoint more representative of the current health condition of veterans with GWI. Using this model, we evaluated the potential for propranolol to reduce the underlying neuroinflammation associated with GWI. We found that this long-term model produced a primed, but dormant neuroinflammatory state that could be elucidated by subsequent immune challenge with the bacterial mimic lipopolysaccharide (LPS). Treatment with propranolol prior to LPS challenge in our long-term model significantly reduced the inflammatory cytokine expression in the brain, suggesting the potential for this therapy to restore healthy neuroinflammatory signaling.

## 2. Materials and methods

### 2.1. Animals

Adult male C57BL/6J mice (*n* = 5–7 mice per group), 8–12 weeks of age were purchased from Jackson Labs (Bar Harbor, ME). All procedures were performed within protocols approved by the Centers for Disease Control and Prevention-Morgantown Institutional Animal Care and Use Committee and the US Army Medical Research and Materiel Command Animal Care and Use Review Office, and the animal facility was certified by AAALAC International. Upon arrival, mice were individually housed in a temperature- (21 ± 1 °C) and humidity-controlled (50% ± 10) colony room that was maintained under filtered positive-pressure ventilation and a 12 h light (0600 EDT)/12 h dark cycle (1800 EDT) and acclimated for one week prior to the commencement of experimental procedures. Mice were given ad libitum access to food (Harlan 7913 irradiated NIH-31 modified 6% rodent chow) and sterile water.

### 2.2. Gulf War Illness exposure paradigm and treatment

**Fig. 1.**
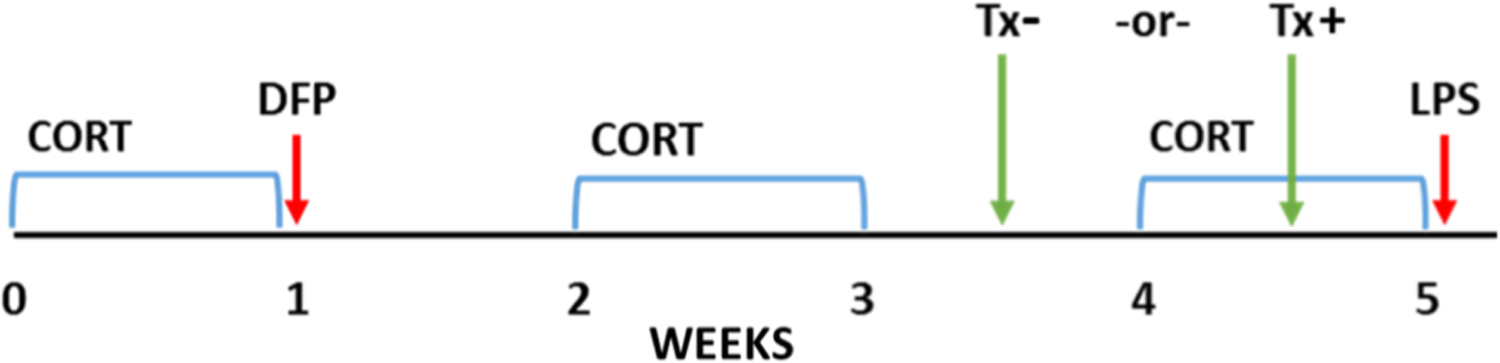
Long-term Gulf War Illness exposure and treatment paradigm. In this exposure paradigm, mice were given corticosterone (CORT) in the drinking water for 7 days followed by a single injection of the sarin surrogate, diisopropyl fluorophosphate (DFP). CORT was then given every other week for a total of 5 weeks. On the final day, mice were challenged with a single injection of lipopolysaccharide (LPS). Propranolol (Tx) was given either during CORT, 4 days prior to LPS challenge (Tx+), or outside of CORT, 11 days prior to LPS challenge (Tx−).

A detailed exposure paradigm is described in Fig. 1. Mice were exposed to CORT (200 mg/L in 0.6% EtOH; Steraloids, Newport, RI, USA) in the drinking water for 7 days. As described previously, this regimen of CORT was chosen due to its ability to achieve circulating levels of stress hormone like those achieved with behavioral repeated stress paradigms, as well as produce significant immunosuppression [7]. On day 7, mice received a single intraperitoneal injection of DFP (4 mg/kg, MilliporeSigma, St. Louis, MO, USA) or physiological saline. To provide additional nutritional support following DFP exposure, all mice were provided with balls of food mash (1:1 ground rodent chow: water) beginning 2 days before exposure (for acclimation) until 2 days following exposure. After the initial exposure, mice received intermittent 7-day bouts of CORT water on days 14–21 and 28–35. On day 35, mice received a single subcutaneous injection of LPS (0.5 mg/kg, MilliporeSigma) or physiological saline. This resulted in 5 groups: Saline, CORT, CORT DFP, CORT LPS, and CORT DFP LPS; this final group receiving all exposures represents our long-term GWI model (GWI group).

For animals receiving treatment, mice were given a single intraperitoneal injection of propranolol (20 mg/kg in saline) either outside of CORT (Day 24; Tx−) or during CORT (Day 31; Tx+). The goal of treatment was to avoid a more direct interaction with either the initiation of the model (i.e. DFP exposure) or the later inflammatory challenge (i.e., LPS). To accomplish this goal, we used the half-life of propranolol to select a time point within our long-term GWI model to administer treatment; we chose to give the drug in the middle of the respective week as propranolol has a relatively short half-life (approximately 4 h). In addition to the GWI groups, propranolol was also given to animals in the CORT LPS group as a treatment control (Supplemental Fig. 1). All animals were sacrificed by decapitation at 6 h following LPS or saline exposure on Day 35.

### 2.3. qRT-PCR

**Fig. 2.**
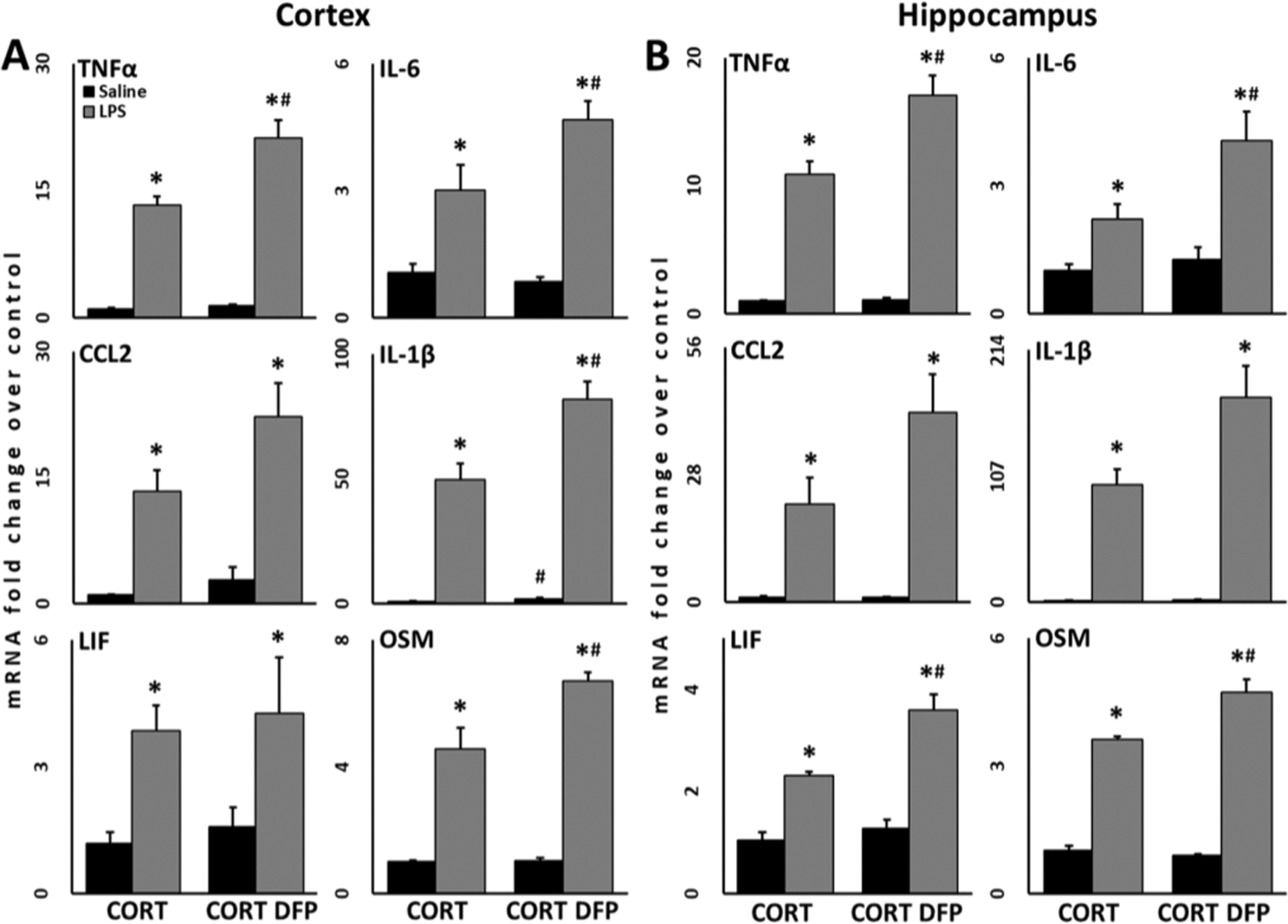
Neuroinflammation is exacerbated in a long-term model of Gulf War Illness. Mice (*N* = 4–7/group) were given corticosterone (CORT) in the drinking water for 7 days followed by a single injection of the sarin surrogate, diisopropyl fluorophosphate (DFP). CORT was then given every other week for a total of 5 weeks. On the final day, mice were challenged with a single injection of lipopolysaccharide (LPS). At 6 h post-LPS, the expression levels of inflammatory cytokine mRNA were measured in the cortex (A) and hippocampus (B). Statistical significance was determined at *p* ≤ 0.05 compared within* or between ^#^ groups (CORT vs CORT DFP).

**Fig. 3.**
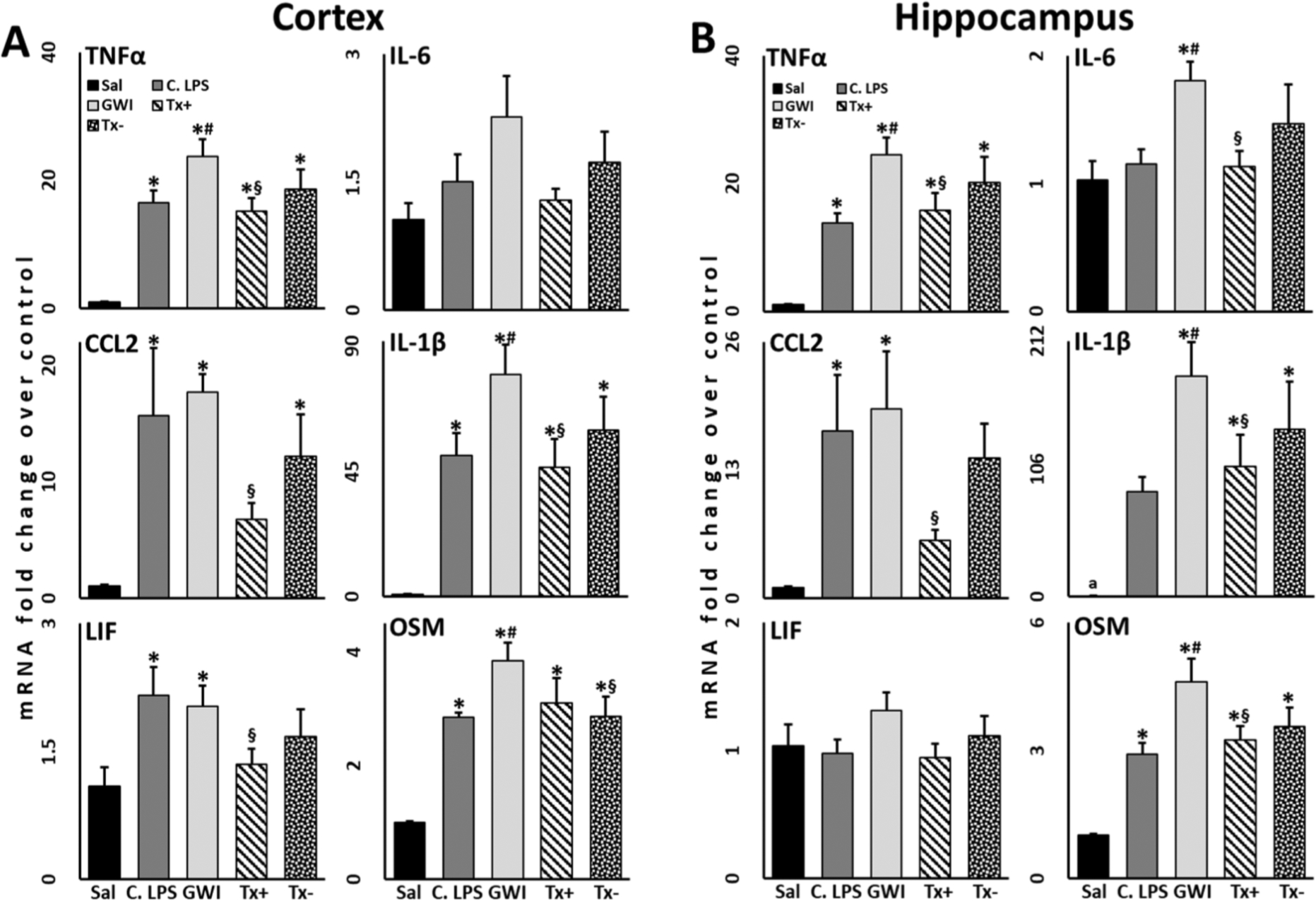
Treatment with propranolol reduces brain inflammatory cytokine expression in a long-term model of GWI. Mice (*N* = 4–7) were given corticosterone (CORT) in the drinking water for 7 days followed by a single injection of the sarin surrogate, diisopropyl fluorophosphate (DFP). CORT was then given every other week for a total of 5 weeks. On the final day, mice were challenged with a single injection of lipopolysaccharide (LPS). Propranolol was administered in the long-term GWI model (CORT DFP LPS) either 4 days (during CORT; Tx+) or 11 days (outside of CORT; Tx−) prior to LPS challenge. At 6 h post-LPS, the expression levels of inflammatory cytokine mRNA were measured in the cortex (A) and hippocampus (B). Statistical significance was determined at *p* ≤ 0.05 compared to saline (Sal)*, CORT LPS (C. LPS)^#^, and GWI^§^. ^a^N = 3.

Immediately after decapitation, whole brains were removed from the skull with the aid of blunt curved forceps. Cortices and hippocampi were dissected free hand on a thermoelectric cold plate (Model TCP-2, Aldrich Chemical Co., Milwaukee, WI, USA) using a pair of fine curved forceps (Roboz, Washington, DC, USA). Brain regions were frozen at −85 °C and used for subsequent isolation of total RNA. Total RNA from cortex and hippocampus was isolated as previously described [7,48]. Real-time PCR analysis of the housekeeping gene, glyceraldehyde-3-phosphate dehydrogenase (GAPDH), and of the proinflammatory mediators, TNFα, IL-6, CCL2, IL-1β, leukemia inhibitor factor (LIF), and oncostatin M (OSM), as well as the astrocyte marker glial fibrillary acidic protein (GFAP) was performed in an ABI7500 Real-Time PCR System (Thermo Fisher Scientific, Waltham, MA, USA) in combination with TaqMan® chemistry as previously described [7,48]. Relative quantification of gene expression was performed using the comparative threshold (ΔΔCT) method. Changes in mRNA expression levels were calculated after normalization to GAPDH. The ratios obtained after normalization are expressed as fold change over corresponding CORT-treated controls for Fig. 2 and saline-treated controls for Fig. 3 and Supplemental Fig. 1.

### 2.4. Statistical analysis

Sample size was calculated previously [7] and determined to be at least *N* = 4/group for a power of 0.8 and α = 0.05. An *N* = 5/group was used for all non-DFP-treated groups to account for the possibility of a statistical outlier and an *N* = 7/group was used for all DFP-treated to account for an approximately 25–30% mortality rate (LD_25_ as determined from historical data) for the dose of DFP administered. Prior to statistical analysis, data were analyzed for outliers using Grubb’s test (α = 0.05) (GraphPad QuickCalcs: https://www.graphpad.com/quickcalcs/Grubbs1.cfm). Statistical analysis of data was performed using one-way or two-way ANOVA on log-transformed values followed by multiple pairwise comparison analysis using Fisher least significant difference (LSD) post hoc test utilizing SigmaPlot (v. 14.5; Systat Software, Inc., San Jose, CA, USA) with statistical significance at 5% (*p* ≤ 0.05). Data were log transformed as they did not follow a normal distribution. For the propranolol treatment data presented in Fig. 2, two separate one-way ANOVA analyses were performed for statistical comparisons of the Tx+ and Tx− propranolol groups to all other conditions, e. g., Sal, CORT LPS, and GWI. Graphical representations are of the mean fold change ± SEM.

### 2.5. Data availability

All data is available on the NIOSH Data and Statistics Gateway (https://www.cdc.gov/niosh/data/).

## 3. Results

### 3.1. Intermittent re-exposure to corticosterone facilitates a long-term, primed neuroinflammatory phenotype in a mouse model of GWI

Our previous work established an acute model of GWI that produced significantly exacerbated neuroinflammation, evidenced by the robust increase in the expression of inflammatory cytokines throughout the brain, when the GWI-relevant, sarin surrogate DFP was given following a subchronic exposure to the stress hormone, CORT [1,7,10,12,13]. This acute paradigm models the exposures that would have been experienced by soldiers deployed in the Gulf War theatre that potentially may be responsible for the initiation of GWI. Here, we have expanded upon this model to better emulate the current condition of veterans with GWI by re-exposing the mice bi-weekly to CORT for 7 days for a total of 5 weeks following the initial CORT DFP exposure (Fig. 1). This paradigm was developed to mimic the intermittent periods of stress that would be experienced by veterans over their daily life following their return from deployment. On its own, re-exposure to CORT does not perpetuate the elevated inflammatory cytokine expression in the brain (Fig. 2, CORT DFP group) as was previously observed acutely following DFP exposure [1,7,10,12,13]. However, when these mice are challenged with an inflammatory stimulus, such as exposure to the bacterial mimic LPS, to model the symptom flare-ups experienced by veterans with GWI following a systemic challenge there is a robust neuroinflammatory response; this is observed in our long-term model as the increased cytokine mRNA expression elicited by CORT DFP LPS exposure that exceeds the levels (approximately 1.5–1.7 fold) produced by CORT LPS in the absence of DFP in both the cortex (Fig. 2A) and the hippocampus (Fig. 2B). Indeed, all the cytokines measured show a statistically significant increase in the GWI model (CORT DFP LPS) over the CORT LPS group except for CCL2 and LIF in the cortex (Fig. 2A) and CCL2 and IL-1β in the hippocampus (Fig. 2B), which demonstrated increases in expression that failed to reach significance. Thus, these data suggest that combined exposure to high physiological stress and GWI-relevant neurotoxicants such as the irreversible acetylcholinesterase inhibitor and sarin surrogate, DFP, can induce an aberrant neuroinflammatory state that can be uncovered by subsequent inflammatory challenges. More specifically, the exposure to GWI-relevant neurotoxicants on a background of high physiological stress results in a significantly more robust priming of the cytokine response to subsequent immune challenge with LPS, compared to the high physiological stress background alone.

While we have demonstrated that our long-term GWI model produces a significantly primed neuroinflammatory response compared to control conditions, e.g. Saline, CORT LPS, and CORT DFP, we wanted to examine how the long-term model compares to our previous findings in our acute GWI model. A comparison of the cytokine mRNA expression profiles for our acute and long-term models illustrates that while both produce significant changes in the brain expression of inflammatory cytokines, the intermittent re-exposure to CORT and challenge with LPS does not produce a universal exaggeration of the responses seen previously in our acute model (Table 1). Interestingly, cortical expression of TNF-α, IL-6, and IL-1β mRNA in our long-term GWI model was significantly increased compared to the previously published data from the acute exposure paradigm, while the expression of LIF and CCL2 in the cortex and hippocampus, respectively, were significantly decreased. Consistent with the dormant neuroinflammatory phenotype we are proposing for our long-term paradigm in the absence of subsequent immune challenge, cytokine mRNA expression in the long-term CORT DFP group was significantly reduced in both the cortex and hippocampus in comparison to the acute GWI model. These findings suggest that the initiating exposures to high physiological stress and GWI-relevant neurotoxicants may produce a persistent, aberrant neuroinflammatory state that could modulate the neuroinflammatory response to subsequent immune challenges.

### 3.2. The beta-adrenergic blocker, propranolol, exerts an anti-inflammatory effect that abrogates the latent neuroinflammatory state present in the long-term GWI mouse model

Our research has continued to support the role of aberrant neuroimmune signaling as an underlying cause of GWI [1,7,10,12,13,30,36,49,50]. However, healthy immune and neuroimmune responses are crucial to general health. As such, we have aimed to identify anti-inflammatory therapeutics that could mitigate or reverse the persistent neuroinflammatory state associated with GWI without instigating prolonged immunosuppression. In order to evaluate this, we can use the CORT LPS condition as a healthy, normal neuroimmune response where immune challenge is instigated following conditions of intermittent stress in the absence of the GWI-relevant chemical exposure. Utilizing this rationale, we found that the β-adrenergic receptor blocker, propranolol, which has been demonstrated to have anti-inflammatory/neuroinflammatory effects, significantly reduced the neuroinflammatory response to LPS challenge in our long-term GWI model, particularly when given during the final week of CORT exposure, four days prior to LPS challenge (Tx+) (Fig. 3). This effect was observed for cytokine mRNAs measured in both the cortex (Fig. 3A) and hippocampus (Fig. 3B). Specifically, propranolol significantly reduced the mRNA expression levels of cortical TNFα, CCL2, IL-1β, LIF, and OSM (Fig. 3A), as well as hippocampal TNFα, IL-6, CCL2, IL-1β, and OSM (Fig. 3B) induced in our GWI model to levels at or below those observed for CORT LPS. While the changes observed for cortical IL-6 (Fig. 3A) and hippocampal LIF (Fig. 3B) were not statistically significant, both of these cytokines also showed a pattern of returning to a level similar to CORT LPS. These observations highlight the potential for this drug to restore healthy, normal neuroimmune function. Interestingly, propranolol treatment exerted no protective effects when given 11 days prior to the LPS challenge (outside of CORT exposure, Tx−) in our long-term GWI model or when given in the CORT LPS condition (Supplemental Fig. 1). Interestingly, when propranolol treatment was given outside of the CORT exposure in the CORT LPS group, most of the inflammatory cytokines measured in the cortex and hippocampus were significantly increased in comparison to CORT LPS alone (Supplemental Fig. 1).

## 4. Discussion

Effective treatments for the constellation of conditions that define GWI are lacking, with the majority of ill veterans receiving treatment for individual symptoms. This is largely due to the fact that the underlying etiology of GWI largely has been unknown for the last several decades. However, there has been a significant increase in preclinical research focused on the discovery of new therapeutics for the treatment of GWI [19,30,51–62]. With recent advances in GWI research pointing to persistent neuroimmune dysfunction as a likely underlying cause of symptoms among ill veterans [1–30], therapeutics that may regulate neuroinflammation are prime candidates for successful GWI treatment. Here, we have demonstrated that our acute model of GWI can be expanded to mimic the chronic condition of GWI that is currently experienced by veterans with GWI by repeatedly and intermittently exposing the mice to CORT after the initiating, “in theatre” exposure paradigm of CORT DFP described by our previously published acute GWI mouse model [1,7,10,12,13]. Furthermore, using this long-term model, we have shown that the β-adrenergic receptor inhibitor and anti-neuroinflammatory drug, propranolol, is capable of mitigating the significant, robust increase in neuroinflammatory cytokine mRNA expression in our long-term model of GWI. Specifically, we found that propranolol treatment appears to remove the GWI-specific neurotoxicant-driven enhanced neuroinflammatory response, returning it to levels comparable to the CORT LPS condition, which we have defined as a “healthy sick” condition.

In our previous work, we established an acute rodent model of GWI to model the in theatre exposure conditions experienced by ill veterans where we combined subchronic CORT exposure with acute exposure to the nerve agent surrogate, DFP, as well as other GWI-relevant pesticide exposures [1,7,10,12,13,36]. This model captured the potential for the high levels of physiological stress experienced during deployment, modeled by exogenous CORT administration, to exacerbate or prime the neuroinflammatory response to exposure to various organophosphate acetylcholinesterase inhibitors that these soldiers may have encountered while in theatre (e.g. sarin and cyclosarin nerve agents; chlorpyrifos and dichlorvos pesticides) [33]. However, veterans with GWI have been ill for over three decades suggesting that these effects persist far beyond their time in theatre. In the current study, we found that intermittent re-exposure to CORT following the DFP exposure, mimicking periods of high stress that would be experienced by veterans upon their return from deployment, perpetuated the priming effect seen in our acute model. This produced a seemingly dormant dysfunctional neuroinflammatory response that resulted in a significantly exacerbated increase in brain cytokine mRNA expression upon inflammatory challenge with LPS, compared to the same paradigm in the absence of DFP exposure (CORT LPS group) (Fig. 2). The cytokine mRNA expression profile observed in our long-term GWI mouse model after LPS challenge at 5 weeks differed from the profile observed in the acute model post-DFP (Table 1). However, the observation that our long-term GWI model produces a nearly consistent fold increase in cytokine mRNA expression across all cytokines and brain areas in comparison to the CORT LPS group, suggests that the GWI-initiating exposures of CORT and DFP produces an underlying neuroinflammatory dysfunction that seems to enhance the inflammatory profile instigated by the later immune challenge. Moreover, this enhancement extends beyond the effects of the stress hormone alone [48]. This observation aligns with the hypothesis that GWI is the result of aberrant homeostatic neuroimmune function [1–30] and resembles the symptom flare-ups reported by veterans with GWI; this will be further explored in our future studies. Additionally, these results lend further support to the neuroimmune hypothesis of GWI by highlighting the persistence of the underlying neuroinflammatory changes, as recently observed in ill veterans via positron emission tomography of the inflammatory molecule TSPO [18], that were likely instigated immediately following the neurotoxic exposures experienced during deployment.

Currently, it is unclear if the neuroimmune dysfunction that has been identified in GWI is the result of direct central inflammation or the instigation of central inflammation by the circulation of peripherally generated cytokines. Indeed, previous studies on sickness behavior have demonstrated that peripherally-induced inflammation by agents such as LPS or the viral mimic, polyinosinic:polycytidylic acid (PIC) results in neuroinflammation [63–67]. Moreover, we have previously shown and substantiated in our current study that our regimen of CORT exposure enhances the neuroinflammatory response to the systemic administration of both LPS and PIC [48]. However, we have found that the priming instigated by the exogenous administration of CORT is seen almost exclusively in the brain, as our CORT exposure regimen largely reduced the inflammatory effects of DFP in the liver and serum [49], as well as the liver of LPS exposed animals [O’Callaghan, unpublished data]. Thus, while we cannot definitively rule out a role for peripheral inflammation in the development of GWI-related neuroinflammation, we believe that the priming of the neuroinflammatory response we observe in both our acute and long-term GWI mouse models is the result of direct effects of CORT and DFP on the brain.

As the etiology of GWI is uncovered, there has been an impetus for the discovery of drugs that are capable of treating GWI itself, as opposed to treating individual or clusters of symptoms. With growing support for the neuroimmune hypothesis of GWI, investigating therapies with the potential to modulate neuroimmune or neuroinflammatory function is crucially important. However, many immunomodulatory therapies have the potential to promote immunosuppression, which is not an ideal outcome for those suffering from GWI. Thus, we developed a goal for our preclinical GWI treatment project that emphasized treating GWI by restoring normal, healthy neuroimmune responses [68,69]. Using our newly established long-term mouse model of GWI, we defined this as a treatment that would recover responses in our GWI group (CORT DFP LPS) to a “healthy sick” state (CORT LPS), since anyone has the potential to experience periods of high physiological stress prior to an inflammatory challenge. For administering potential therapeutics, we aimed to time the treatment in a manner that avoided interfering with the initiation of GWI in our model (i.e. initial CORT DFP exposure). We also wanted to avoid treating the known inflammatory effects of the LPS challenge. In other words, the objective was to treat an already present illness, one defined by aberrant neuroinflammatory signaling, by resetting and modulating the potentially exacerbated responses to likely inflammatory challenges. Here, we found that treatment with propranolol days prior to immune challenge with LPS appears to mitigate the neuroinflammatory consequences of the Gulf War-specific organophosphate exposure, reducing the observed fold increase of cytokine mRNA expression between GWI and CORT LPS groups and, in many cases, returned the expression of the cytokines to CORT LPS, “healthy sick” levels (Fig. 3). While we found a difference in the effectiveness of propranolol treatment at our two treatment timepoints, it is unclear whether this is a result of the timing of administration (4 days vs 11 days prior to LPS) or the presence or absence of CORT at the time of injection, and what impact this could potentially have on an ill veteran receiving treatment. Moreover, while treatment with propranolol 4 days prior to LPS in the CORT LPS “healthy sick” group had largely no effect on cytokine mRNA expression, we did observe that treatment 11 days pre-LPS in this group paradoxically exacerbated the neuroinflammatory responses (Supplemental Fig. 1). These findings suggest that the timing of treatment administration is an important aspect to consider in future studies, both preclinical and clinical, when evaluating propranolol as a candidate therapy in the treatment of GWI.

Our treatment effect with propranolol suggests the potential for a novel therapeutic for GWI. The effects of propranolol demonstrated here are similar to what has been shown previously in studies of other illnesses with a neuroinflammatory component. Wohleb and colleagues [38] found that treatment with propranolol reduced the expression of inflammatory markers on microglia and macrophages, as well as reduced microglial IL-1β mRNA expression in their mouse model of stress-induced anxiety and depression. Additionally, propranolol was found to reduce TNFα and GFAP protein, among other inflammatory markers, in a rat model of ischemia [41]. However, in both of these studies, propranolol was given as a pre-treatment prior to their experimental procedure. Thus, to the best of our knowledge, our study is the first to demonstrate a post-exposure anti-inflammatory effect of propranolol. While propranolol has been demonstrated to have anti-neuroinflammatory effects, it remains unclear as to the direct mechanism by which propranolol exerts its effects and warrants further investigation.

## 5. Conclusion

We have shown that our acute, in theatre exposure mouse model of GWI can be expanded into a long-term GWI model and presents an ideal paradigm to evaluate the ability of a candidate drug to treat the neuroinflammatory response associated with GWI and return that response to a healthy state. Our results suggest that propranolol may be an effective treatment for GWI due to its ability to modulate and reduce the primed neuroinflammatory response observed in our long-term GWI mouse model that is believed to be associated with the illness, while showing the potential to avoid the suppression of a healthy immune response. Overall, these positive results seen with propranolol suggest that anti-neuroinflammatory drugs show promise as potential therapeutics for treating the underlying neuroimmune dysfunction that has become associated with GWI.

## Supplementary Material

supplemental figure

## Figures and Tables

**Table 1:** Comparison of cytokine mRNA fold change expression over control (±SEM) between acute and long-term Gulf War Illness mouse models.

	Acute Model^a^		Long-term Model				Long-term GWI v. Acute CORT DFP	
	CORT DFP		CORT DFP		GWI			
	Cortex	Hippocampus	Cortex	Hippocampus	Cortex	Hippocampus	Cortex	Hippocampus
**TNF-α**	12.75±3.07	32.33±10.90	1.40±0.22	1.04±0.15	21.23±2.16	17.58±1.76	**↑8.48** *	**↓14.75**
**IL-6**	1.55±0.30	4.92±1.58	0.85±0.10	1.50±0.34	4.67±0.46	4.22±0.82	**↑3.12** *	**↓0.7**
**CCL2**	73.70±27.58	138.17±43.16	2.80±1.55	1.62±0.65	22.25±3.96	43.12±10.10	**↓51.45**	**↓95.05** *
**IL-1β**	25.85±6.39	295.85±91.61	2.05±0.51	1.48±0.27	81.96±6.96	183.63±30.56	**↑56.11** *	**↓112.22**
**LIF**	45.43±22.37	27.63±12.49	1.59±0.44	1.13±0.14	4.26±1.33	3.73±0.32	**↓41.17** *	**↓23.9**
**OSM**	6.49±1.85	8.34±2.19	1.03±0.10	0.89±0.05	6.72±0.27	4.81±0.35	**↑0.23**	**↓3.53**

a Data values from O’Callaghan et al. [1].

* p ≤ 0.05.

## Abbreviations:

CORT: corticosterone
DFP: diisopropyl fluorophosphate
GAPDH: glyceraldehyde-3-phosphate dehydrogenase
GWI: Gulf War Illness
LIF: leukemia inhibitory factor
LPS: lipopolysaccharide
OSM: oncostatin M
PIC: polyinosinic:polycytidylic acid
