# Supplementary material for: The β-adrenergic receptor blocker and anti-inflammatory drug propranolol mitigates brain cytokine expression in a long-term model of Gulf War Illness
Source: Life Sci. Author manuscript; Available in PMC 2022 Apr 28. (PMC9047058; doi:10.1016/j.lfs.2021.119962)

**The β-adrenergic receptor blocker and anti-inflammatory drug propranolol mitigates brain cytokine expression in a long-term model of Gulf War Illness**

Lindsay T. Michalovicz^a^, Kimberly A. Kelly^a^, Diane B. Miller^a^, Kimberly Sullivan^b^, James P. O’Callaghan^a*^

^a^Health Effects Laboratory Division, Centers for Disease Control and Prevention-National Institute for Occupational Safety and Health, Morgantown, WV, USA

^b^School of Public Health, Boston University, Boston, MA, USA

^*^Corresponding Author

James P. O’Callaghan, PhD

1000 Frederick Ln, M/S L3014

Morgantown, WV 26508

(304) 285-6079

[Jdo5@cdc.gov](mailto:Jdo5@cdc.gov)

**Supplemental Figure 1. Propranolol treatment did not ameliorate neuroinflammation in the absence of DFP.** Mice (N=4-7) were given corticosterone (CORT) in the drinking water for 7 days every other week for a total of 5 weeks. On the final day, mice were challenged with a single injection of lipopolysaccharide (LPS). Propranolol was given either 4 days (during CORT; Tx+) or 11 days (outside of CORT; Tx-) prior to LPS challenge in the CORT LPS (C. LPS) exposure condition. At 6 hours post-LPS, the expression levels of inflammatory cytokine mRNA were measured in the cortex (A) and hippocampus (B). Statistical significance was determined at p≤0.05 compared to saline (Sal)* and C. LPS^#^.


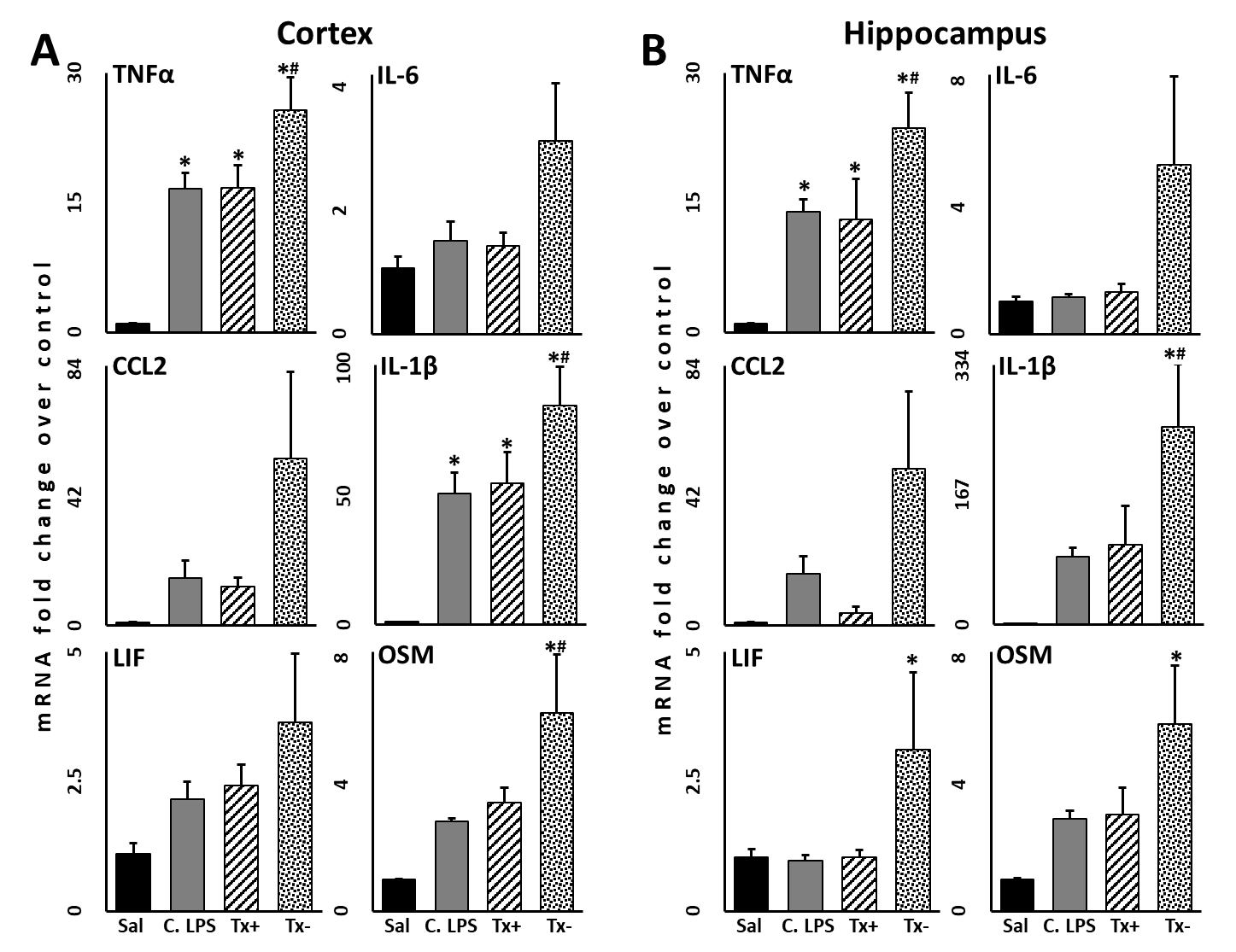

Supplement: supplemental figure [file NIHMS1793699-supplement-supplemental_figure.docx]
